# *Prunus pananensis* (Rosaceae), a New Species from Pan'an of Central Zhejiang, China

**DOI:** 10.1371/journal.pone.0054030

**Published:** 2013-01-18

**Authors:** Zi-Lin Chen, Wei-Jie Chen, Huan Chen, Ying-Ying Zhou, Mei-Qin Tang, Meng-Qi Fu, Xiao-Feng Jin

**Affiliations:** 1 College of Life & Environment Sciences, Hangzhou Normal University, Hangzhou, Zhejiang, People's Republic of China; 2 Zhejiang Dapanshan National Natural Reserve, Pan'an, Zhejiang, People's Republic of China; 3 Zhejiang Institute of Microbiology, Hangzhou, Zhejiang, People's Republic of China; University College London, United Kingdom

## Abstract

*Prunus pananensis* Z. L. Chen, W. J. Chen & X. F. Jin, a new species of Rosaceae from central Zhejiang, China is described and illustrated. Micromorphological characters of the indumentum on young shoots, leaves, petioles and peduncles, including scanning electron microscope [SEM] images, are provided. This new species is morphologically similar to *P. schneiderianae* Koehne in having its young shoots, petioles and pedicels all densely villose, but differs in having bracts persistent, styles glabrous, stipules 8–9 mm long, stamens 28–30 of per flower, and drupes glabrous. The new species is also similar to *P. discoidea* (Yü & C. L. Li) Yü & C. L. Li ex Z. Wei & Y. B. Chang in having 2 or 3 flowers in an umbellate inflorescence, and bracts persistent and marginally glandular, but it differs in having young shoots and petioles densely covered with yellowish-brown villose trichomes; leaves rounded or slightly cordate at base, the mid-ribs and lateral veins abaxially densely covered with yellowish-brown villose trichomes; and hypanthium ca. 3 mm long, shorter than sepals. The *atp*B-*rbc*L and *trn*L-F intergenic chloroplast spacers are selected for identification of the new and its similar species.

## Introduction

Fruit type, carpel numbers and ovary position are frequently used for the traditional classification of the family Rosaceae. Four subfamilies, Spiraeoideae, Rosoideae, Maloideae and Prunoideae, were proposed. This subfamilial classification of Rosaceae is currently in a state of flux, and most systematists no longer recognize the Prunoideae, which is instead now included within an expanded Spiraeoideae [1]. Spiraeoideae is now replaced by Maloideae.

*Prunus* L. (s.l.), containing 430 species, is mainly distributed in the northern hemisphere, especially in the temperate zone [2]–[4]. The woody genus *Prunus* s.l. has sometimes been segregated into several genera: *Amygdalus* L., *Armeniaca* Scop., *Cerasus* Mill., *Laurocerasus* Tourn. ex Duh., *Padus* Mill. and *Prunus* s.s. [5], [6]. Alternatively, these segregate genera are often treated as subgenera or sections within *Prunus* [4], [7]–[9]. Phylogenetic analysis of molecular data has shown that *Cerasus*, *Laurocerasus*, and *Padus* are not monophyletic, supporting the traditional subgeneric classification of Rehder [10], [11].

Subg. *Cerasus* (Mill.) Focke, comprising ca. 40 species, includes deciduous fruit trees and garden ornamentals and is mainly distributed in eastern Asia [4], [6]. Koehne proposed a system of subg. *Cerasus* worldwide, and two groups (not ranked) with four sections, 14 subsections and several series were established [7]. Although *Cerasus* is no longer recognized at the generic rank in most floras, this subgenus has been treated as an independent genus with several sections in China. There are 44 species of subg. *Cerasus* in China, and the independent genus *Cerasus* was used by Li and Bartholomew [6]. Yü and Li proposed a generic system of the Chinese species, with two subgenera and 11 sections recognized [5], [6].

During 2010 to 2012, the senior author (Jin) organized botanical trips in Dapanshan National Natural Reserve and the adjacent regions of Pan'an County, Zhejiang Province, China. In these trips, previously undescribed species of *Prunus* in sect. *Lobopetalum* (Koehne) Yü & C. L. Li was collected. This species is morphologically similar to *P. schneiderianae* Koehne in having young shoots, petioles and pedicels that are all densely villose, but differs in having bracts persistent, styles and drupes glabrous, stipules 8–9 mm long, and stamens 28–30 of per flower. The species is also similar to *P. discoidea* (Yü & C. L. Li) Yü & C. L. Li ex Z. Wei & Y. B. Chang in having 2 or 3 flowers in umbellate inflorescence, and bracts persistent and glandular at margin, but differs in having young shoots and petioles densely pubescent with yellowish-brown villose trichomes; leaves rounded or slightly cordate at base, their mid-ribs and lateral veins abaxially densely covered with yellowish brown villose trichomes; and hypanthium ca. 3 mm long, shorter than sepals. Based on studies on morphology and DNA barcoding, we concluded that it represents a new species, described below.

## Materials and Methods

### Taxon sampling

The new species and its putative relatives, *Prunus discoidea* and *P. schneideriana*, were sampled for this study. *Prunus serrulata*, the species grew together with *Prunus discoidea* and *P. schneideriana* in eastern China, was selected as the outgroup. *Prunus serrulata* also has spreading sepals, petals 2-lobed, drupes purplish black and inflorescences subumbellate, but its bracts are deciduous. Fresh leaves were collected from a total of 16 individuals from different populations in Zhejiang and Anhui (Table 1). All voucher specimens were deposited in HTC (Herbarium of Hangzhou Normal University, formerly Herbarium of Hangzhou Teachers' College). The approvals of the sampling were obtained of Administration of Zhejiang Dapanshan National Natural Reserve (China), Administration of Zhejiang Qingliangfeng National Natural Reserve (China), Administration of Tonglingshan National Forestry Park (China) and Administration of Anhui Qiyunshan Forestry Park (China).

**Table 1:** Sources of materials sequenced and their GenBank numbers.

Taxon	Locality	Voucher	GenBank accession numbers (*atp*B–*rbc*L)	GenBank accession numbers (*trn*L–*trn*F)
*P. discoidea*				
1	Liukou, Xiuning, Anhui, China	*X. F. Jin 2746*	JX847385	JX847401
2	Liangyuan, Changhua, Zhejiang, China	*X. F. Jin 2020*	JX847386	JX847402
3	Shuangxi, Pan'an, Zhejiang, China	*X. F. Jin 2679*	JX847387	JX847403
4	Huzhai, Pan'an, Zhejiang, China	*X. F. Jin 2654*	JX847388	JX847404
5	Dazhekeng, Pan'an, Zhejiang, China	*X. F. Jin 2660*	JX847389	JX847405
6	Dazhekeng, Pan'an, Zhejiang, China	*X. F. Jin 2661*	JX847390	JX847406
7	Dazhekeng, Pan'an, Zhejiang, China	*X. F. Jin s. n.*	JX847391	JX847407
*P. pananensis*				
1	Huaxi, Pan'an, Zhejiang, China	*X. F. Jin & al. 2651*	JX847379	JX847395
2	Huaxi, Pan'an, Zhejiang, China	*X. F. Jin & al. 2540*	JX847380	JX847396
3	Gao'er, Pan'an, Zhejiang, China	*X. F. Jin & al. 2658*	JX847381	JX847397
4	Niuluxi, Pan'an, Zhejiang, China	*X. F. Jin & al. s. n.*	JX847382	JX847398
5	Antian, Pan'an, Zhejiang, China	*X. F. Jin & al. 2686*	JX847383	JX847399
*P. schneideriana*				
1	Mt. Tongling, Wencheng, Zhejiang, China	*X. F. Jin 2846*	JX847392	JX847408
2	Mt. Tongling, Wencheng, Zhejiang, China	*X. F. Jin 2776*	JX847393	JX847409
3	Shiyang, Wencheng, Zhejiang, China	*X. F. Jin 2814*	JX847394	JX847410
*P. serrulata*	Mt. Tongling, Wencheng, Zhejiang, China	*X. F. Jin 2697*	JX847384	JX847400

### SEM observation

The indumentum on young shoots, leaves, petioles and peduncles of *Prunus discoidea*, *P. schneideriana* and *P. pananensis* was observed using a Philips XL-30E scanning electron microscope (SEM). The sampled materials were cleaned in 50% ethanol for 30 min, and air dried. The cleaned materials were mounted on stubs using double-sided adhesive tape, and sputter-coated with gold prior to SEM observations.

### DNA extraction, PCR and sequencing

Samples for DNA extraction were dried in silica gel. Total genomic DNA was extracted using standard CTAB method [12]. The PCR amplifications were carried out on a DNA Engine PCR (Bio-Rad) in 50 µL reactions, and two chloroplast DNA regions (namely *atp*B–*rbc*L and *trn*L–*trn*F) were used [13]. Each reaction contained 5.0 µL 10× buffer, 2.0 µL dNTPs (2 mmol/µL), 1.0 µL each primer (10 mmol/µL), 4.0 µL genomic DNA (20 ng), 0.5 µL Taq polymerase (5 U/µL), and 36.5 µL ddH_2_O. The PCR program began at 94°C for 1 min, followed by 34 cycles of 94°C for 30 s, 50°C for 30 s and 72°C for 2 min, followed by a 72°C extension for 5 min [13]. All PCR products were electrophoresed on 1% agarose gel to verify product size.

PCR products were purified with AxyPrep PCR Clean-up Kit (Axygen, China) following the manufacturer's instructions. Sequencing was carried out using PCR primers on an ABI 3730 automated sequencer (AppliedBiosystems, USA).

### Data analysis

The non-coding regions have relatively higher evolution rates, as well as more informative characters, and are typically more useful in identifying species than are coding regions [13]–[15]. As recommended by Quan and Zhou, *atp*B-*rbc*L and *trn*L-*trn*F were selected as DNA barcoding regions for species identification in *Prunus*. The edited data matrix was analyzed using ClustalX 1.83 to obtain an initial multiple alignment, keeping default alignment parameters [16]. Phylogenetic relationships were analyzed with PAUP* 4.0b10 for maximum parsimony (MP) and MrBayes v.3.0b4 for Bayesian inference (BI), respectively [17], [18]. Both MP tree and Bayesian tree were conducted for the concatenated data of *atp*B-*rbc*L and *trn*L-*trn*F sequences, and gaps were treated as zero (“missing”). For the MP analysis, a heuristic search algorithm with 1000 random addition replicates and tree bisection and reconnection (TBR) branch-swapping. Node support was assessed using 1000 MP bootstrap (BS) replicates. Using Akaike information criterion in Modeltest 3.7, the most appropriate model of sequence evolution for BI was estimated [19]. The analyses were conducted with four Metropolis-coupled Markov chains in each of two independent runs of 10^7^ generations. A 50% majority-rule consensus tree was computed by PAUP* after “burn-in”, and the posterior probabilities (PP) were calculated.

## Results

### Indumentum on young shoots, petioles, leaves and pedicels

**Figure 1:**
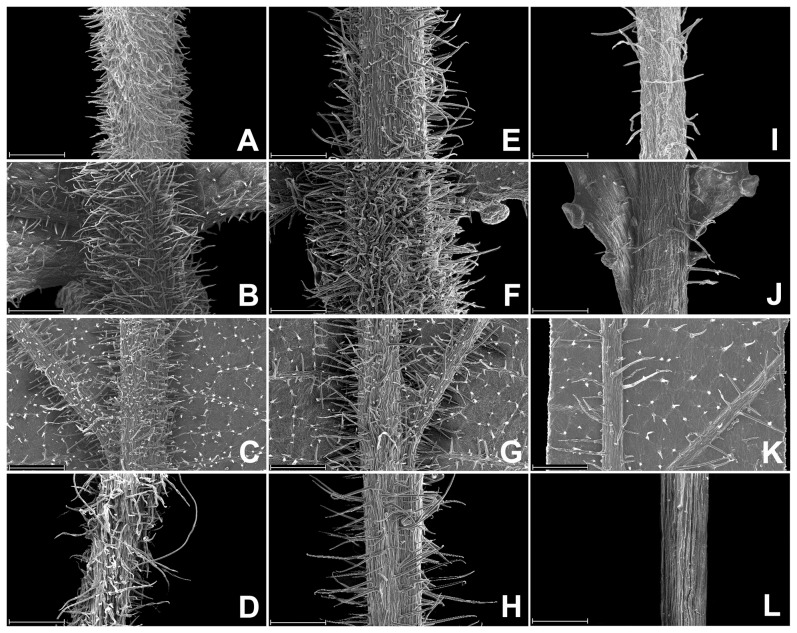
SEM photographs of the indumentum on young shoots, petioles, leaves and pedicels of *Prunus pananensis*, *P. schneideriana* and *P. discoidea*. A–D. *P. pananensis*; E–H. *P. schneideriana*; I–L. *P. discoidea*. A, E, I. Indumentum on young shoots; B, F, J. Indumentum on petioles; C, G, K. Indumentum on costa and lateral vein of abaxial leaf surfaces; D, H, L. Indumentum on pedicels. Scale bars = 1 mm.

The indumentum on young shoots, petioles, leaves and pedicels is shown in Figure 1. The indumentum on young shoots and petioles of the new species *Prunus pananensis* and the similar species *P. schneideriana* is densely villose (Fig. 1: A, B, E, F). The leaves of these two species were villose on costa and lateral veins abaxially (Fig. 1: C, G), and their pedicels were also villose (Fig. 1: D, H). In contrast, the young shoots and petioles of *P. discoidea* were sparsely villose (Fig. 1: I, J), and the leaves were sparsely villose on costa and lateral veins abaxially (Fig. 1: K). The pedicels of *P. discoidea* were glabrous (Fig. 1: L).

### DNA barcoding/molecular phylogenetic analysis

The combined *atp*B-*rbc*L and *trn*L-*trn*F dataset consisted of 17 individuals belonging to four species, and included 1717 aligned characters. The *atp*B-*rbc*L and *trn*L-*trn*F contained 787 and 930 characters, respectively. For *atp*B-*rbc*L, 4 nucleotide positions were variable and 5 were parsimony-informative. For *trn*L-*trn*F, 56 were variable and 6 were parsimony-informative. Maximum parsimony produced a MP tree with 72 steps.

**Figure 2:**
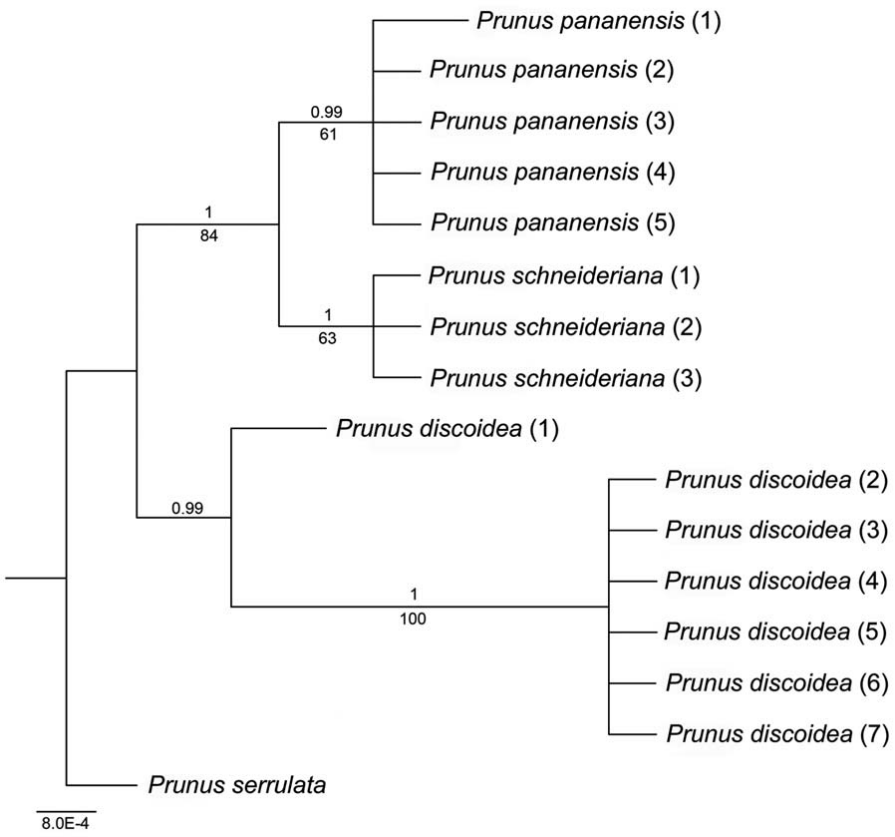
Majority consensus tree infered from Bayesian analysis based on the *atp*B-*rbc*L and *trn*L-F data. Numbers above the branches are posterior probabilities (PP≥50%); numbers below the branches are maximum parsimony bootstrap percentage (BS≥50%).

Bayesian analysis produced a tree was not significantly different from the MP tree, and the Bayesian tree was shown in Figure 2. The five individuals of *Prunus pananensis* formed a clade that is sister to *P. schneideriana*. The clade of *P. pananensis*+*P. schneideriana* was well supported with 84% boostrap support, and thus *P. pananensis* is supported as more closely related to *P. schneideriana* than to *P. discoidea*.

The molecular evidence indicated that the individuals of *Prunus pananensis* formed an independent clade and fit the phylogenetic species concept [20]–[22]. Further, the new species and the sister species are reciprocally monophyletic. The morphological observations showed that *Prunus pananensis* also fit the diagnosable species concept and the traditional phenetic/morphological species concepts [1].

## Conclusion

### Taxonomic treatment

**Figure 3:**
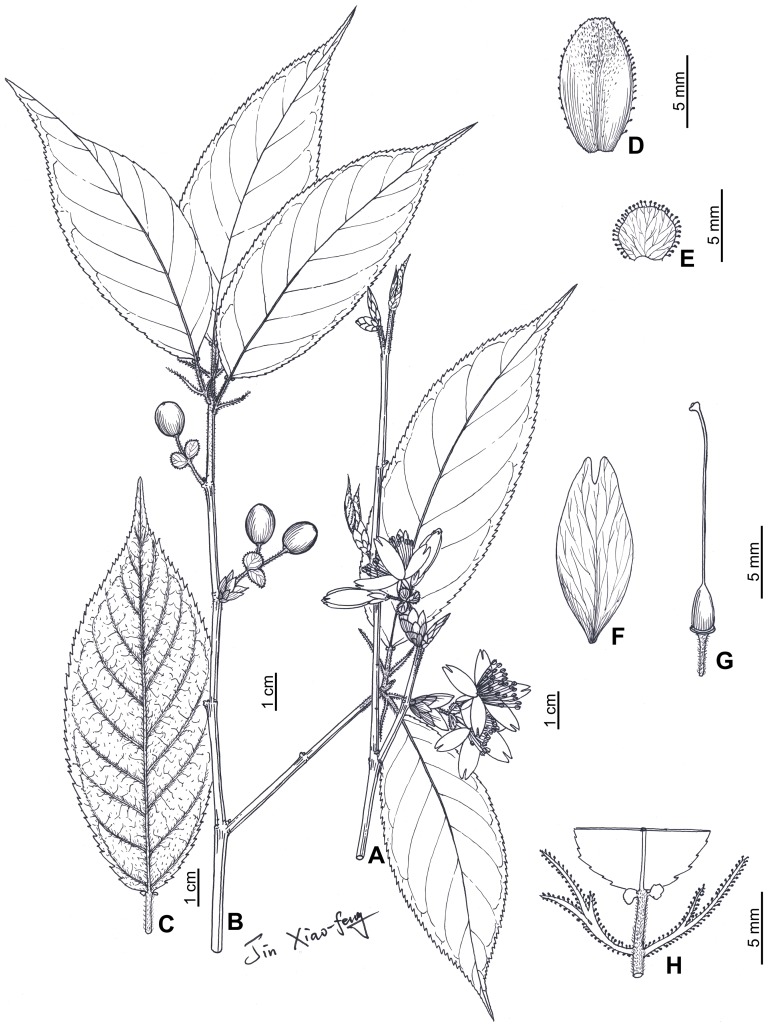
*Prunus pananensis* Z. L. Chen, W. J. Chen & X. F. Jin. **A.** Flowering shoot; **B.** Fruiting shoot; **C.** Leaf, showing adaxial indumentums; **D.** Involucral bract; **E.** Bract; **F.** Petal; **G.** Pistil; **H.** Leaf base, showing stipules.

**Prunus pananensis Z. L. Chen, W. J. Chen & X. F. Jin, sp. nov. [urn:lsid:ipni.org:names:77124052-1] (** **Figures 3** **and** **4** **). Type:** —CHINA. Zhejiang Province: Pan'an County, Dapanshan National Natural Reserve, Huaxi Valley, Xiaolongtan, in forests along valley, 29°00′17.20″N, 120°29′45.38″E, elevation 470 m, 30 March 2011 (fl.), *X. F. Jin & Z. L. Chen 2651* (holotype HTC!, isotype ZJFC!, ZM!).

**Figure 4:**
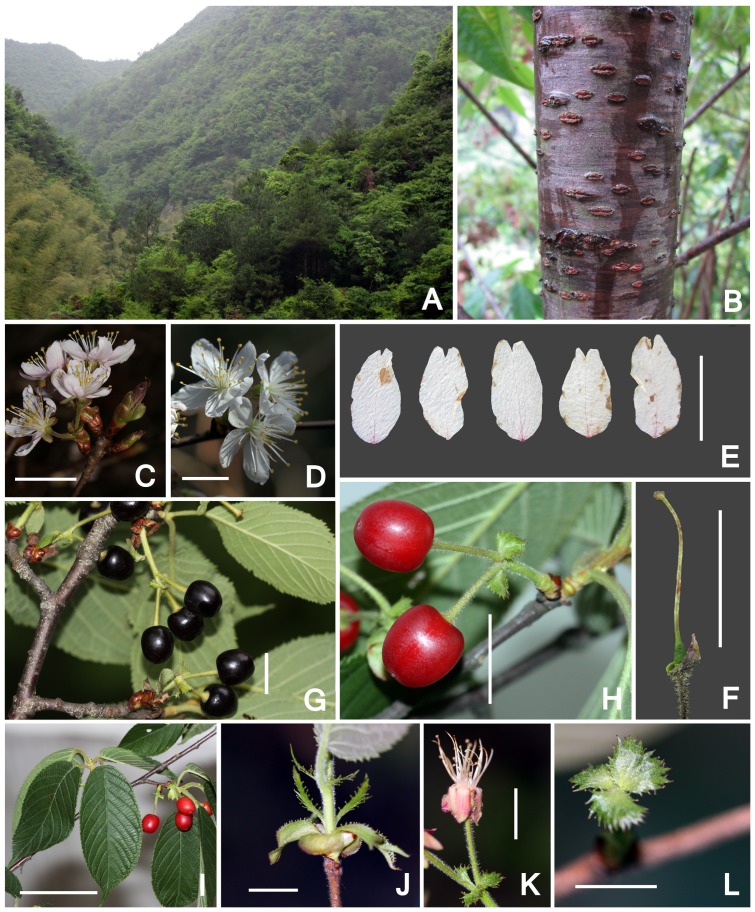
Photographs of *Prunus pananensis* Z. L. Chen, W. J. Chen & X. F. Jin. **A.** Natural habitat of the Huaxi Valley in Mt. Dapan, Pan'an, China; **B.** Bark with lenticels; **C.** Pink flowers; **D.** White flowers; **E.** Petals, showing the shape and 2-lobed apex; **F.** Pistil, showing glabrous ovary, style and capitate stigma; **G.** Fruiting shoot, showing purplish fruits; **H.** Red fruits; **I.** Leaves, showing the shape; **J.** Stipule, showing glandular margin; **K.** A flower without petals, showing reflexed sepals; **L.** Bracts, showing the shape and glandular margin. Scale bars: 2 cm in C, 1 cm in D–H, J–L, 5 cm in I.

Heac species *P. schneiderianae* Koehne affinis, sed bracteis persistentibus, stylis glabris, stipulis 8–19 mm longis, staminibus 28–30 differt. Species etiam affinis *P. discoideae* (Yü & C. L. Li) Yü & C. L. Li ex Z. Wei & Y. B. Chang, sed ramulis petiolisque fulvo-villosis, foliis basi rotundatis vel leviter cordatis, costis mediis et nervis lateralibus subtus fulvo-villosis, tubo calycino lobis calycinis breviore, circ. 3 mm longo differt.

Trees deciduous, 2.4–8 m tall. Bark brown, with grayish brown or grayish white lenticels. Young shoot densely covered with yellowish brown villose trichomes, becoming brown-pubescent later. Winter buds ovoid, glabrous. Leaf blades obovate-elliptic, elliptic or oblong, chartaceous, 4–10(–12) cm long, 2–4.5(–6) cm wide, caudate, rarely acuminate at apex, rounded or slightly cordate at base, margin acutely serrulate and teeth with a minute disciform apical gland, adaxially greenish yellow to yellowish brown (when dried), almost glabrous, abaxially brown (when dried), and costa and lateral veins densely covered with yellowish brown villose trichomes; lateral veins 8–10 pairs, curved. Petiole 6–12 mm long, densely covered with yellowish brown villose trichomes, with one pair of glands at apex. Stipules narrowly linear, 8–19 mm long, deeply lobed, margin pectinate and teeth with a minute conical apical gland. Inflorescences umbellate, 2 or 3-flowered, rarely solitary, with scales at base; scales leathery, purplish brown, broadly ovate, 3–5 mm long, 2.5–4 mm wide, glabrous on both surfaces; involucral bracts green, apex brown, obovate, 6–8 mm long, 5–6 mm wide, obtuse and serrate-lobed at apex, margin with minute glands, adaxially hirsute, densely at upper part, abaxially glabrous; peduncles 6–10 mm long, concealed in scales or slightly exserted, spreading pilose; bracts green, suborbicular, 3–4 mm in diam., leathery, sparsely pilose, margin with minute disciform glands; pedicels 8–12 mm long, spreading pilose. Flowers opening before or with leaves; hypanthium campanulate, ca. 3 mm long, 2–2.5 mm wide, pilose; sepals ovate-oblong, reflexed, 5–6.5 mm long, 2–3 mm wide, acuminate or acute at apex, outside sparsely pilose at margin, inside glabrous; petals pink or white, ovate-elliptic to elliptic-oblong, 11–15 mm long, 5–7.5 mm wide, 2-lobed at apex; stamens 28–30, filaments 5–12 mm long, unequal, anthers broadly ovoid; ovary ovoid, glabrous, ca. 2.5 mm long; style glabrous, 10–12 mm long; stigma capitate. Drupes globose, red or purplish black at maturity, 7–8 mm in diam., glabrous; endocarp slightly sculptured. Fl. & Fr. Mar.-May.

**Etymology:** — The species is name after Pan'an County in Zhejiang Province, eastern China.

**Additional Collections (paratypes):** — CHINA. Zhejiang Province: Pan'an County, Dapanshan National Natural Reserve, Huaxi Valley, Xiaolongtan, in forests along valley, 29°00′17.20″N, 120°29′45.38″E, elevation ca. 470 m, 30 March 2011 (fl.), *X. F. Jin & Z. L. Chen 2649, 2650* (HTC, ZM); Dapanshan National Natural Reserve, Huaxi Valley, Dafengkeng, 28°59′37.86″N, 120°30′08.08″E, elevation ca. 680 m, 25 April 2010 (fr.), *X. F. Jin, S. F. Xu & W. J. Chen 2540, 2541* (HTC, ZM); Dapanshan National Natural Reserve, Niuluxi, elevation 550 m, 25 May 2010, *X. F. Jin & L. Qian s. n.* (HTC, ZM), the same locality, 26 May 2010, *X. F. Jin & T. T. Shen s. n.* (HTC, ZJFC, ZM); Gao'er Township, Mount Gaomu, 28°56′28.86″N, 120°39′17.37″E, elevation ca. 950 m, 10 April 2011 (fr.), *X. F. Jin 2658* (HTC, ZM); Antian Township, Shiliutian, 28°58′50.69″N, 120°35′29.26″E, elevation 511 m, 20 April 2011 (fr.), *X. F. Jin & W. J. Chen 2685, 2686, 2687* (HTC, ZJFC).

**Nomenclature:** — The electronic version of this article in Portable Document Format (PDF) in a work with an ISSN or ISBN will represent a published work according to the International Code of Nomenclature for algae, fungi, and plants [23], [24], and hence the new names contained in the electronic publication of a PLOS ONE article are effectively published under that Code from the electronic edition alone, so there is no longer any need to provide printed copies.

In addition, new names contained in this work have been submitted to IPNI, from where they will be made available to the Global Names Index. The IPNI LSIDs can be resolved and the associated information viewed through any standard web browser by appending the LSID contained in this publication to the prefix http://ipni.org/. The online version of this work is archived and available from the following digital repositories: PubMed Central, LOCKSS.
